# Sex differences in the association between metabolic disorder and inflammatory cytokines in Han Chinese patients with chronic schizophrenia

**DOI:** 10.3389/fpsyt.2024.1520279

**Published:** 2025-01-03

**Authors:** Yinghan Tian, Zhenkuo Li, Yun Zhang, Pei Tang, Yu Zhuang, Lewei Liu, Haojie Fan, Xianhu Yao, Wenzheng Li, Lei Xia, Huanzhong Liu

**Affiliations:** ^1^ Department of Psychiatry, Chaohu Hospital of Anhui Medical University, Hefei, Anhui, China; ^2^ Anhui Psychiatric Center, Anhui Medical University, Hefei, Anhui, China; ^3^ Department of Psychiatry, The Fifth People’s Hospital of Xiangtan City, Xiangtan, China; ^4^ Department of Psychiatry, School of Mental Health and Psychological Sciences, Anhui Medical University, Hefei, Anhui, China; ^5^ Department of Psychiatry, Ma’anshan Fourth People’s Hospital, Ma’anshan, Anhui, China; ^6^ Department of Psychiatry, Hefei Fourth People’s Hospital, Hefei, Anhui, China; ^7^ Anhui Provincial Key Laboratory for Brain Bank Construction and Resource Utilization, Hefei, Anhui, China

**Keywords:** metabolic disorder, inflammatory cytokines, IL-1β, schizophrenia, sex differences

## Abstract

**Background:**

Previous evidence suggests that immune-inflammatory dysfunction plays an important role in metabolic disorder (MD) of patients with schizophrenia, yet studies on sex differences in the association between both are limited. The current study aimed to investigate sex differences in the association between MD and inflammatory cytokines in Han Chinese patients with chronic schizophrenia (CS).

**Methods:**

This multicentre cross-sectional study was conducted in one general hospital and two psychiatric hospitals in Anhui Province, China. General information, metabolic parameters and inflammatory cytokine indicators were collected from all participants, and the severity of their psychiatric symptoms was assessed using the Positive and Negative Syndrome Scale (PANSS).

**Results:**

A total of 324 patients completed the assessment and were included in this study. The patients with MD had higher age and duration of illness, and lower chlorpromazine equivalents and negative symptom score than those without MD. Binary logistic regression showed that MD was significantly associated with a higher IL-1β level in male patients, while this association was not significant in females. Further multiple linear regression revealed that IL-1β level was negatively correlated with polypharmacy only in male patients.

**Conclusion:**

Sex differences in the association between MD and IL-1β level are significant in patients with CS, and only in male patients is there a negative correlation between MD and IL-1β level. Sex-specific prevention and intervention strategies may contribute to reducing MD in patients with CS.

## 1 Introduction

Schizophrenia is a severe and chronic psychotic disorder characterized by heterogeneous domains of thought, perception, behavior and cognition, which affects approximately 24 million people globally (1). Compared with the general population, patients with schizophrenia have a 2-3 times higher risk of premature mortality, primarily due to cardiovascular problems resulting from metabolic disorder (MD) (2, 3).

Patients with schizophrenia often suffer from obesity, hypertension, hyperglycaemia/diabetes and hyperlipidaemia. Having one or more of these conditions is considered to have MD (4) while having three or more is considered to have metabolic syndrome (MetS) (5). Two recent cross-sectional studies both indicated that more than 60% of patients with schizophrenia had comorbid MD (4, 6). Another meta-analysis reported that the prevalence of MetS in people with schizophrenia was 33.4%, significantly surpassing the rate in healthy controls (7). In addition to increased risks of cardiovascular disease and death, individuals with metabolic abnormalities also experience significant declines in cognitive function and quality of life (8, 9). In patients with schizophrenia, MD may have a negative impact on sleep quality and medical comorbidities, leading to a worse disease prognosis (5, 10). Several risk factors may explain the comorbidity of schizophrenia and MD, such as aging, poor dietary patterns, sedentary lifestyle and side effects of antipsychotics (11). Furthermore, other intricate factors are thought to be involved in the onset and progression of MD, including genetic susceptibility (12), sex hormone (13) and oxidative stress imbalance (14), of which immune-inflammatory dysfunction is currently considered to be a crucial mechanism for the comorbidity of MD in schizophrenia (15).

Cytokines, primarily secreted by immune cells, are key molecules in regulating inflammation and are also involved in schizophrenia and MD pathophysiology. On the one hand, previous studies have demonstrated that pro-inflammatory cytokines are elevated at various levels in patients with schizophrenia. A meta-analysis of 24,921 participants revealed that consistently elevated levels of interleukin (IL)-1β, IL-6, and tumor necrosis factor (TNF)-α in patients with acute and chronic schizophrenia (CS) compared with healthy controls (16). An early study found that IL-1β, IL-6 and TNF-α could inhibit cortical neuronal dendritic development and possibly increase the risk of schizophrenia (17). In addition, the IL-17 family is the newest subclass of cytokines, and a recent case-control study suggested that elevated IL-17A level may serve as a potential biomarker for schizophrenia (18). On the other hand, studies have shown that in both animals and humans, higher levels of inflammatory cytokines are significantly associated with a higher risk of MetS. For example, an animal study showed that long-term administration of olanzapine may cause insulin resistance (IR), by significantly increasing levels of IL-1β, IL-6 and TNF-α (19), and a prospective study with 12-month follow-up found that schizophrenics with a higher baseline IL-6 level had an increased risk of subsequent MetS (20).

Moreover, sex-specific associations between schizophrenia and MD have been identified in certain studies. For example, Zeng et al. documented a significantly higher prevalence of MetS in females (13.4%) than in males (6.6%) of patients with schizophrenia (21). Additionally, a previous study found that Body mass index (BMI) was negatively associated with the Positive and Negative Symptom Scale (PANSS) total score and the negative symptom subscale score in male individuals with schizophrenia, but not in females (22). This suggests that there may be sex differences in the associations between metabolic parameters and psychopathology. Also, sex differences have been found in inflammation levels among a variety of neuro-psychiatric disorders, including Alzheimer’s disease (23), depression (24), and schizophrenia (25). Two cross-sectional studies of patients with schizophrenia showed that elevated inflammatory cytokine levels may be associated with more severe psychopathology and poorer sleep quality in females, but not in males (25, 26).

Therefore, this study aimed (1) to investigate the associations between MD and inflammatory cytokines levels, and (2) whether there were sex differences in these associations, and (3) to further analyze the influencing factors of inflammatory cytokines in patients of different sexes with CS. We hypothesize that there will be sex differences in the associations between MD and inflammatory cytokines levels in patients with CS.

## 2 Methods

### 2.1 Study design and participants

This multicentre, cross-sectional study was conducted in one general hospital (Chaohu) and two psychiatric hospitals (Hefei and Ma’anshan) in Anhui Province, China, from May to December 2018. Patients were included, if they met the following criteria: (1) Han Chinese, aged between 18 and 75 years; (2) diagnosed with schizophrenia, based on the Diagnostic and Statistical Manual of Mental Disorders, 5th edition (DSM-5) using a structured Clinical Interview; (3) duration of illness was more than 5 years (27). Patients were excluded who had (1) any other psychiatric disorders; (2) infectious or immune system diseases that may alter the inflammatory state of the body, or other serious physical diseases; (4) or were pregnant or breastfeeding women; (3) recently used any non-steroidal anti-inflammatory drugs, corticosteroids or other immunomodulatory agents.

All participants and their guardians signed informed consent forms in accordance with the Declaration of Helsinki. The protocol of this study was approved by the Medical Ethics Committee of Chaohu Hospital of Anhui Medical University (201805-kyxm-03) and was registered in the Chinese Clinical Trial Registry (ChiCTR1800017044).

### 2.2 Data collection measurements

#### 2.2.1 Socio-demographic and clinical characteristics

Socio-demographic and clinical data were collected via a predesigned questionnaire, including age, sex, marital status, education level, exercise, smoking, antipsychotic medications, age of onset and duration of illness. The dose of antipsychotics per patient was converted to chlorpromazine equivalents using the Defined Daily Dose (DDD) conversion method recommended by the World Health Organization (28). Psychotic symptoms of the patients were assessed by two experienced psychiatrists using the Chinese version of the 30-item PANSS (29, 30), and the correlation coefficients of repeated assessments was more than 0.8 between the scorers. The scale consists of three subscales, including a positive symptom subscale, a negative symptom subscale and a general psychopathology subscale, with higher scores indicating more severe symptoms (31).

#### 2.2.2 Metabolic parameters and inflammatory cytokines levels measurements

Height and weight were measured with patients dressed lightly and with their shoes removed. BMI was calculated as weight (kg)/height (m)^2^. Blood samples were collected from patients between 07:00 and 08:00 AM after an overnight fast. The samples were numbered, centrifuged at 3,600 rpm for 10 minutes, and separated into serum. The serum was immediately stored at -80°C and tested within 30 days. Fasting blood glucose (FBG), triglyceride (TG) and high density lipoprotein-cholesterol (HDL-C) levels were measured by the oxidase method (Meikang Biotechnology Company, Zhejiang, China), the GPO-PAP method (Beijing Lidan Biochemical Company, Beijing, China) and the terminal method (Neusoft Whitman Biotechnology Company, Jiangsu, China), respectively. For inflammatory cytokines, IL-1β, IL-6, IL-17 and TNF-α levels were measured using the sandwich enzyme-linked immunosorbent assay (Sangon Biotech Company, Shanghai, China). The inflammatory cytokines levels were log-transformed as the Kolmogorov-Smirnov test showed that they did not follow a normal distribution.

#### 2.2.3 MD evaluation basis

According to the criteria recommended by the Chinese Diabetes Society (32), individuals with MD have one or more of the following items: (1) BMI ≥ 25.0 kg/m^2^; (2) FPG ≥ 6.1 mmol/L and/or 2-hour postprandial glucose ≥ 7.8 mmol/L and/or have been diagnosed with diabetes and treatment; (3) systolic blood pressure/diastolic blood pressure ≥ 140/90 mmHg (1 mmHg = 0.133 kPa), and/or have been diagnosed with hypertension and treatment; and (4) TG ≥ 1.7 mmol/L, and/or HDL-C < 0.9 mmol/L (male) or HDL-C < 1.0 mmol/L (female).

### 2.3 Statistical analysis

The continuous variables were described as mean ± standard deviation (SD) and the categorical variables were described as frequency distributions (%). The Kolmogorov-Smirnov test was used to test the normal distribution of continuous variables. To compare sex differences in MD among patients with schizophrenia, a two-way analysis of covariance (ANCOVA) was performed to test the effects of diagnosis (with and without MD) and sex (male and female). The main effects of diagnosis and sex, and the interaction of diagnosis*sex were examined. Binary logistic regression models (Forward: LR) were used to examine the independent factors associated with MD in male and female patients, with MD as the dependent variable, and the variables which were significant in the ANCOVA (*P* < 0.05) as the independent variables (e.g., age, duration of illness, chlorpromazine equivalents, and negative symptom score). Forward selection is a stepwise regression method that starts with an empty model, adds variables one by one, and selects the best predictor variables from the available options by fine-tuning the model. The correlations between inflammatory cytokines levels and other clinical data in the patients were examined with Pearson or Spearman correlation analyses. Then multivariate linear regression models (Forward: LR) were used to examine any significant correlations (*P* < 0.05) in correlation analyses. Statistical Product and Service Solutions (SPSS) version 23.0 (SPSS incorporated, Chicago, Illinois, United States of America) was used for statistical analyses. The *P* - values were set as two-tailed α = 0.05.

## 3 Results

### 3.1 Participant characteristics

A total of 324 patients completed the assessment and were included in the statistical analyses. As shown in **Table 1**, the mean age of the patients was 45.10 years (SD = 11.67) and the mean duration of illness was 18.99 years (SD = 10.47), with 192 males (59.3%) and 132 females (40.7%). Of these patients, more than half (56.8%) were being treated with polypharmacy, and 252 (77.8%) had comorbid MD.

**Table 1:** Socio-demographic and clinical characteristics of patients.

Variables	Total sample (*N* = 324)
	N (%)
Male	192 (59.3)
Marital status	
Single	231 (71.3)
Married	93 (28.7)
Exercise	78 (24.1)
Smoking	125 (38.6)
Antipsychotic medications	
Monotherapy	140 (43.2)
Polypharmacy	184 (56.8)
Metabolic disorder	252 (77.8)
	Mean (SD)
Age (years)	45.10 ± 11.67
Education (years)	8.07 ± 3.62
Age of onset (years)	26.12 ± 8.30
Duration of illness (years)	18.99 ± 10.47
Chlorpromazine equivalents (mg/d)	455.81 ± 258.37
PANSS total score	77.96 ± 24.34
Positive symptom score	17.95 ± 7.50
Negative symptom score	21.53 ± 7.63
General psychopathology score	38.48 ± 12.76
Metabolic parameters	
BMI (kg/m^2^)	24.10 ± 3.84
Systolic blood pressure (mmHg)	114.78 ± 12.24
Diastolic blood pressure (mmHg)	75.94 ± 8.11
FBG (mmol/L)	5.36 ± 1.37
TG (mmol/L)	2.24 ± 1.52
HDL-C (mmol/L)	1.05 ± 0.27
Inflammatory cytokines	
Log IL-1β (pg/mL)	0.46 ± 0.46
Log IL-6 (pg/mL)	0.01 ± 0.53
Log IL-17 (pg/mL)	0.72 ± 0.59
Log TNF-α (pg/mL)	1.30 ± 0.23

PANSS, Positive and Negative Symptom Scale; BMI, body mass index; FBG, fasting blood glucose; TG, triglyceride; HDL-C, high-density lipoprotein cholesterol; IL, interleukin; TNF, tumor necrosis factor; SD, standard deviation.

### 3.2 Sex differences in socio-demographic and clinical characteristics between groups with and without MD

As shown in **Table 2**, patients in the MD group had higher age (*F* = 4.81, *P* = 0.029) and duration of illness (*F* = 4.07, *P* = 0.045), and lower chlorpromazine equivalents (*F* = 6.01, *P* = 0.015) and negative symptom score (*F* = 4.17, *P* = 0.042) than those in the non-MD group. In addition, there were significant differences in the positive symptom score (*F* = 4.19, *P* = 0.042) and IL-1β level (*F* = 6.09, *P* = 0.014) in the “diagnosis*sex” interaction. Further simple effects analyses found that in the MD group, male patients had a higher positive symptom score than female patients, and in the males, patients with MD had a higher IL-1β level than those without MD (all *P* < 0.05).

**Table 2:** Socio-demographic and clinical characteristics of male and female patients with schizophrenia with and without MD.

Variables	MD		Non-MD		Diagnosis		Sex		Diagnosis*sex	
	Male (*N* = 145)	Female (*N* = 107)	Male (*N* = 47)	Female (*N* = 25)	*F/χ2*	*P*	*F/χ2*	*P*	*F*	*P*
Age (years)	45.08 ± 11.53	46.88 ± 11.29	42.60 ± 12.66	42.28 ± 11.35	4.806	**0.029**	0.210	0.647	0.427	0.514
Education (years)	8.37 ± 3.31	8.25 ± 3.93	6.70 ± 3.46	8.20 ± 3.91	2.937	0.088	1.913	0.168	2.589	0.109
Single/Married	115/34	63/44	37/10	16/9	0.242	0.622	14.265	**<0.001**	–	–
Exercise (yes/no)	31/114	29/78	12/35	6/19	0.043	0.835	0.726	0.394	–	–
Smoking (yes/no)	91/54	5/102	29/18	0/25	0.113	0.737	113.792	**<0.001**	–	–
Age of onset (years)	25.81 ± 7.66	26.76 ± 8.46	25.55 ± 9.29	26.20 ± 9.46	0.125	0.724	0.471	0.493	0.016	0.898
Duration of illness (years)	19.43 ± 10.37	19.81 ± 10.75	17.30 ± 10.01	16.08 ± 10.48	4.066	**0.045**	0.083	0.773	0.301	0.584
Chlorpromazine equivalents (mg/d)	405.09 ± 220.97	480.47 ± 270.41	502.18 ± 296.50	557.30 ± 284.25	6.013	**0.015**	3.385	0.067	0.082	0.775
Monotherapy/Polypharmacy	66/79	44/63	22/25	8/17	0.090	0.764	1.322	0.250	–	–
PANSS total score	81.26 ± 25.02	71.94 ± 24.03	82.66 ± 21.64	75.72 ± 22.10	0.598	0.440	5.909	**0.016**	0.126	0.722
Positive symptom score	19.10 ± 7.86^*^	16.47 ± 6.94	17.32 ± 7.61	18.92 ± 6.62	0.104	0.747	0.250	0.617	4.186	**0.042**
Negative symptom score	21.99 ± 7.33	19.50 ± 7.38	25.49 ± 7.82	20.20 ± 7.17	4.167	**0.042**	14.246	**<0.001**	1.843	0.176
General psychopathology score	40.19 ± 13.38	36.04 ± 12.56	39.85 ± 11.27	36.60 ± 11.25	0.004	0.949	4.399	**0.037**	0.065	0.799
Metabolic parameters										
BMI (kg/m^2^)	24.43 ± 3.84	25.59 ± 3.67	20.92 ± 2.14	21.85 ± 2.42	55.581	**<0.001**	4.610	**0.033**	0.053	0.817
Systolic blood pressure (mmHg)	114.94 ± 10.76	117.23 ± 13.93	109.06 ± 11.10	114.04 ± 11.64	7.365	**0.007**	4.720	**0.031**	0.646	0.422
Diastolic blood pressure (mmHg)	76.33 ± 8.28	77.13 ± 8.13	72.66 ± 7.36	74.76 ± 6.93	7.342	**0.007**	1.692	0.194	0.340	0.560
FBG (mmol/L)	5.43 ± 1.39	5.60 ± 1.63	4.88 ± 0.53	4.73 ± 0.52	14.407	**<0.001**	0.001	0.970	0.756	0.385
TG (mmol/L)	2.45 ± 1.47	2.64 ± 1.75	1.20 ± 0.30	1.25 ± 0.30	44.572	**<0.001**	0.347	0.556	0.127	0.722
HDL-C (mmol/L)	0.95 ± 0.24	1.07 ± 0.24	1.17 ± 0.26	1.31 ± 0.25	46.147	**<0.001**	14.26	**<0.001**	0.137	0.711
Inflammatory cytokines										
Log IL-1β (pg/mL)	0.49 ± 0.45^+^	0.44 ± 0.47	0.33 ± 0.31^*^	0.59 ± 0.61	0.004	0.949	2.791	0.096	6.092	**0.014**
Log IL-6 (pg/mL)	-0.001 ± 0.51	0.04 ± 0.57	-0.04 ± 0.46	0.06 ± 0.58	0.011	0.917	0.875	0.350	0.150	0.699
Log IL-17 (pg/mL)	0.66 ± 0.57	0.78 ± 0.58	0.69 ± 0.61	0.84 ± 0.71	0.343	0.559	2.733	0.099	0.041	0.840
Log TNF-α (pg/mL)	1.30 ± 0.21	1.32 ± 0.28	1.29 ± 0.13	1.32 ± 0.29	0.005	0.942	0.565	0.453	0.015	0.901

MD, metabolic disorder; PANSS, Positive and Negative Symptom Scale; BMI, body mass index; FBG, fasting blood glucose; TG, triglyceride; HDL-C, high-density lipoprotein cholesterol; IL, interleukin; TNF, tumor necrosis factor; Bolded *P* values < 0.05.

The asterisk (*) indicates the comparison between males and females with schizophrenia with or without MD.

The plus sign (+) indicates the comparison between schizophrenics with and without MD in males and females.

### 3.3 Factors associated with MD in male and female patients

In male patients, binary logistic regression analysis found that MD was significantly associated with lower chlorpromazine equivalents [odds ratio (OR) = 0.999, 95% confidential interval (CI) = 0.997-1.000] and negative symptom score (OR = 0.939, 95% CI = 0.896-0.984), and a higher level of IL-1β (OR = 2.719, 95% CI = 1.052-7.026) (all *P* < 0.05) (**Table 3**). Unfortunately, there were no significant differences in socio-demographic and clinical characteristics between the groups in female patients (all *P* > 0.05).

**Table 3:** Independent factors associated with MD in male patients.

Variables	*P*	OR	95% CI	
			Lower	Upper
Chlorpromazine equivalents (mg/d)	**0.048**	0.999	0.997	1.000
Negative symptom score	**0.008**	0.939	0.896	0.984
Log IL-1β (pg/mL)	**0.039**	2.719	1.052	7.026

IL, interleukin; OR, odds ratio; CI, confidential interval; Bolded *P* values < 0.05.

### 3.4 Sex differences in correlations between IL-1β level and other clinical variables

The results were shown in **Table 4**. In male patients, IL-1β level was negatively correlated with duration of illness (*r* = -0.172, *P* = 0.017) and polypharmacy (*r* = -0.183, *P* = 0.011), and was positively correlated with MD (*r* = 0.163, *P* = 0.024) and level of TG (*r* = 0.195, *P* = 0.007). However, there were no significant correlations between IL-1β level and other clinical variables in female patients (all *P* > 0.05). Further multivariate linear regression analyses showed that in male patients, IL-1β level was negatively correlated with polypharmacy (*β* = -0.185, *P* = 0.009, R^2^ = 0.061) and positively correlated with MD (*β* = 0.165, *P* = 0.020, R^2^ = 0.061).

**Table 4:** Correlations between IL-1β level and other clinical variables in male and female patients.

Variables	Male		Female	
	*r*	*P*	*r*	*P*
Age (years)	-0.133	0.067	-0.166	0.057
Education (years)	0.041	0.576	0.060	0.491
Single/Married	-0.090	0.215	-0.142	0.104
Exercise (yes/no)	-0.091	0.211	-0.088	0.317
Smoking (yes/no)	-0.008	0.916	-0.001	0.996
Age of onset (years)	-0.055	0.450	-0.134	0.126
Duration of illness (years)	-0.172	**0.017** ^a^	-0.119	0.173^a^
Monotherapy/Polypharmacy	-0.183	**0.011**	0.044	0.620
Chlorpromazine equivalents (mg/d)	-0.094	0.192	-0.016	0.853
Metabolic disorder (yes/no)	0.163	**0.024** ^a^	-0.119	0.175^a^
PANSS total score	0.097	0.182	-0.092	0.292
Positive symptom score	0.108	0.136	-0.046	0.597
Negative symptom score	0.015	0.838	-0.147	0.093
General psychopathology score	0.107	0.140	-0.064	0.465
Metabolic parameters				
BMI (kg/m^2^)	0.054	0.456	0.016	0.858
Systolic blood pressure (mmHg)	-0.023	0.753	-0.097	0.271
Diastolic blood pressure (mmHg)	-0.117	0.107	-0.119	0.174
FBG (mmol/L)	-0.027	0.713	0.007	0.938
TG (mmol/L)	0.195	**0.007** ^a^	0.125	0.152^a^
HDL-C (mmol/L)	-0.063	0.383^a^	0.073	0.407^a^

PANSS, Positive and Negative Symptom Scale; BMI, body mass index; FBG, fasting blood glucose; TG, triglyceride; HDL-C, high-density lipoprotein cholesterol; a, Spearman correlation analysis; Bolded *P* values < 0.05.

## 4 Discussion

In this study, we investigated the associations between MD with clinical variables and inflammatory cytokines, and their sex differences. Subsequently, the factors influencing inflammatory cytokines in patients with CS of different sexes were further analyzed. Consistent with previous studies in both healthy populations and clinical patients, we observed that age and duration of illness were significant risk factors for MD (33, 34). For instance, a prospective study of patients with recent-onset psychosis reported that age was an effective predictor of changes in FBG level (34). In addition, Zhang et al. found that ageing had a significant effect on other components of MD besides impaired FBG (35). It is important to note that duration of illness is not only associated with age, but also reflects the degree of antipsychotic exposure in patients with CS, both of which are related to an increased risk of MD (36).

We also found the negative associations between MD with negative symptoms and chlorpromazine equivalents in patients with CS. The current evidence on the association between MD and negative symptoms is controversial. Previous studies of first-episode and chronic patients with schizophrenia found that negative symptoms were negatively associated with BMI (37, 38), while other studies reported opposite results. Two studies from the United States and Spain indicated that negative symptoms were positively associated with MetS and IR in patients with schizophrenia (39, 40). In this study, the patients with MD had a lower negative symptom score, possibly due to their better efficacy and response to antipsychotics. A recent meta-analysis showed that clozapine and olanzapine, although prone to metabolic side effects, were significantly better than other antipsychotics at improving psychiatric symptoms in patients with schizophrenia (41). Additionally, several neuroimaging studies suggested that dysregulated reward system function may mediate the association between MD and negative symptoms in patients with schizophrenia. For instance, a recent study found that a higher risk of weight gain with antipsychotics was associated with a greater connectivity between reward-related regions and other functional regions of the brain (42). Another cross-sectional study from Switzerland showed that scores on the apathy dimension of negative symptoms in schizophrenia were negatively correlated with ventral striatal functional connectivity, which plays an important role in reward processes (43). Therefore, the patients with lower negative symptoms may have less impaired reward responsiveness and may be more likely to use food intake as an available reward cue, whereas uncontrolled food consumption ultimately leads to MD (37). Moreover, negative symptoms may affect the chlorpromazine equivalents in patients with CS, and collectively are involved in the regulation of metabolism. However, our results should be interpreted with caution given that the OR values in this study are modest. Further studies are needed to confirm whether these statistically significant results also carry clinical significance across different races, regions, and phases of illness in patients.

This study also found that there were significant interactions between MD and sex on positive symptoms and IL-1β level. First, male patients had a higher positive symptom score than female patients in the MD group, but not in the non-MD group. Sex differences have been a topic of great interest in schizophrenia research, and brain imaging studies have shown that there may be differences in the functioning of brain regions in male and female patients. Salehi et al. suggested that sex differences in schizophrenia may influence the interaction between positive symptoms and temporal cortex activation (44). Another longitudinal study found that among first-episode psychosis patients, males and those with more severe positive symptoms were more likely to be resistant to treatment, suggesting that they may have residual psychiatric symptoms during the chronic course of the illness (45). An additional possible explanation is that MD exacerbates the severity of positive symptoms in male but not female schizophrenics, which may be related to the neuroprotective effects of estrogen, including anti-inflammatory and anti-oxidative stress (46, 47).

Second, we found a positive association between MD and IL-1β level in male patients with CS. Similarly, previous studies have shown that MD was associated with elevated level of IL-1β both in the general population and in patients with schizophrenia (48, 49). IL-1β is a major pro-inflammatory cytokine secreted mainly by monocytes/macrophages, and its elevated levels indicate that the body is in a state of immune-inflammatory activation. The current view suggests that MD is associated with a chronic low-grade inflammatory state, and that elevated levels of pro-inflammatory cytokines may lead to IR or other metabolic comorbidities (50). On the one hand, an animal study found that IL-1β could promote adipogenesis by directly targeting adipocyte precursors (51). On the other hand, apart from maintaining energy homeostasis, adipose tissue has been shown to have endocrine regulatory properties, releasing a variety of inflammatory cytokines and chemokines that induce chronic low-grade inflammation (52). In addition, obesity promotes the transition of macrophages to the M1 phenotype, which secretes more pro-inflammatory cytokines (e.g., TNF-α, IL-6, and IL-1β) (53). Overall, increased IL-1β level may be both a cause and a consequence of MD, whereas the exact mechanism has not been clarified and further studies are needed to explore the associations between them. Notably, we found that there was a positive correlation between MD and IL-1β level only in male patients. Similarly, Fang et al. discovered that patients with schizophrenia in the MetS group had a significant higher level of IL-6 than those in the non-MetS group, and that this difference was only found in males (54). Interestingly, Bosia et al. also found a significant interaction of IL-1β genotype with MetS and sex in cognitive function, with T-carrier females with MetS performing worse in executive function (55). To the best of our knowledge, few systematic studies have examined sex differences between MD and inflammatory cytokine levels in patients with schizophrenia, in which sex hormones may play a moderating role. Imano et al. observed that a pro-inflammatory environment appeared earlier in male mice than in females during the high-fat diet-induced obesity process, which may be related to the regulation of senescence-associated T cells by estrogen (56). Furthermore, studies have shown that estradiol and progesterone may also indirectly reduce IL-1β level by enhancing the production of prostaglandin E2 (57). Therefore, our findings indicate that male patients may experience more pronounced immune-inflammatory responses under MD conditions, which could contribute to a greater risk of physical disease.

Finally, we further examined the factors influencing IL-1β level in patients with CS, and the multivariate regression analyses showed that the IL-1β level was negatively correlated with polypharmacy only in male patients. In patients with schizophrenia, antipsychotics could reduce levels of proinflammatory cytokines to improve immune-inflammatory activation. A meta-analysis of 43 studies reported an average 23.16% reduction in IL-1β level after risperidone treatment, particularly in patients with CS (58). In contrast, Zhao et al. reported that BMI, FBG level and IL-1β level were significantly increased after clozapine administration in patients with schizophrenia, and positive associations were found between IL-1β level and daily dose of clozapine (59). Thus, taking into account drug interactions, metabolic side effects, the impact on inflammation, and heterogeneity in response to treatment, our results should be considered preliminary.

Some limitations were noted in this study. Firstly, this study was cross-sectional design, the results were not able to address the direction of causality between MD and IL-1β level. Therefore, future studies could consider a longitudinal design to better assess their causal relationships. Secondly, this study included only inpatients with CS, and further research could enrol patients with different phases to explore deeper. Thirdly, this study did not measure the sex hormone levels of the participants and did not assess the menstrual status of the female patients, which may affect our further interpretation of the results. Finally, the patients included had been treated with different antipsychotics. Although these antipsychotics were converted to equal doses of chlorpromazine, this may not have eliminated all possible confounding effects.

## 5 Conclusion

In conclusion, age and duration of illness are significant risk factors for MD, and patients with MD have less severe negative symptoms. The results also show that there are significant sex differences in the association between MD and IL-1β level in patients with CS, with the negative association between MD and IL-1β levels only in males. These findings provide new insights into the inflammatory immune regulation of metabolism in schizophrenia and might help direct further research into this important aspect. Given the clinical utility, regular monitoring of metabolic parameters and inflammatory cytokine levels will be necessary.

## Data Availability

The original contributions presented in the study are included in the article/supplementary material. Further inquiries can be directed to the corresponding author.

## References

[1] Solmi M Seitidis G Mavridis D Correll CU Dragioti E Guimond S. Incidence, prevalence, and global burden of schizophrenia - data, with critical appraisal, from the Global Burden of Disease (GBD) 2019. Mol Psychiatry. (2023) 28:5319–27. doi: 10.1038/s41380-023-02138-4 37500825

[2] Ali S Santomauro D Ferrari AJ Charlson F. Excess mortality in severe mental disorders: A systematic review and meta-regression. J Psychiatr Res. (2022) 149:97–105. doi: 10.1016/j.jpsychires.2022.02.036 35259666

[3] Polcwiartek C O'Gallagher K Friedman DJ Correll CU Solmi M Jensen SE. Severe mental illness: cardiovascular risk assessment and management. Eur Heart J. (2024) 45:987–97. doi: 10.1093/eurheartj/ehae054 PMC10972692 38538149

[4] Peng XJ Hei GR Li RR Yang Y Liu CC Xiao JM. The association between metabolic disturbance and cognitive impairments in early-stage schizophrenia. Front Hum Neurosci. (2020) 14:599720. doi: 10.3389/fnhum.2020.599720 33692676 PMC7937877

[5] Yan H Huang Z Lu Y Qiu Y Li M Li J. Associations between metabolic disorders and sleep disturbance in patients with schizophrenia. Compr Psychiatry. (2023) 122:152369. doi: 10.1016/j.comppsych.2023.152369 36702060

[6] Garrido-Torres N Ruiz-Veguilla M Alameda L Canal-Rivero M Ruiz MJ Gómez-Revuelta M. Prevalence of metabolic syndrome and related factors in a large sample of antipsychotic naïve patients with first-episode psychosis: Baseline results from the PAFIP cohort. Schizophr Res. (2022) 246:277–85. doi: 10.1016/j.schres.2022.07.007 35878542

[7] Vancampfort D Stubbs B Mitchell AJ De Hert M Wampers M Ward PB. Risk of metabolic syndrome and its components in people with schizophrenia and related psychotic disorders, bipolar disorder and major depressive disorder: a systematic review and meta-analysis. World Psychiatry. (2015) 14:339–47. doi: 10.1002/wps.20252 PMC4592657 26407790

[8] Hagi K Nosaka T Dickinson D Lindenmayer JP Lee J Friedman J. Association between cardiovascular risk factors and cognitive impairment in people with schizophrenia: A systematic review and meta-analysis. JAMA Psychiatry. (2021) 78:510–8. doi: 10.1001/jamapsychiatry.2021.0015 PMC7931134 33656533

[9] Chen SH Chen SC Lai YP Chen PH Yeh KY. Abdominal obesity and hypertension are correlated with health-related quality of life in Taiwanese adults with metabolic syndrome. BMJ Open Diabetes Res Care. (2020) 8:e000947. doi: 10.1136/bmjdrc-2019-000947 PMC7039578 32079613

[10] Teixeira AL Martins LB Berk M Bauer ME. Severe psychiatric disorders and general medical comorbidities: inflammation-related mechanisms and therapeutic opportunities. Clin Sci (Lond). (2022) 136:1257–80. doi: 10.1042/CS20211106 36062418

[11] Saccaro LF Aimo A Panichella G Sentissi O. Shared and unique characteristics of metabolic syndrome in psychotic disorders: a review. Front Psych. (2024) 15:1343427. doi: 10.3389/fpsyt.2024.1343427 PMC10944869 38501085

[12] Espeso-Gil S Halene T Bendl J Kassim B Ben Hutta G Iskhakova M. A chromosomal connectome for psychiatric and metabolic risk variants in adult dopaminergic neurons. Genome Med. (2020) 12:19. doi: 10.1186/s13073-020-0715-x 32075678 PMC7031924

[13] Goh KK Chen CY-A Wu T-H Chen C-H Lu M-L. Crosstalk between schizophrenia and metabolic syndrome: the role of oxytocinergic dysfunction. Int J Mol Sci. (2022) 23:7092. doi: 10.3390/ijms23137092 35806096 PMC9266532

[14] Hertiš Petek T Marčun Varda N. Childhood cardiovascular health, obesity, and some related disorders: insights into chronic inflammation and oxidative stress. Int J Mol Sci. (2024) 25:9706. doi: 10.3390/ijms25179706 39273654 PMC11396019

[15] Foiselle M Barbosa S Godin O Wu C-L Boukouaci W Andre M. Immuno-metabolic profile of patients with psychotic disorders and metabolic syndrome. Results from the FACE-SZ cohort. Brain Behav Immun Health. (2022) 22:100436. doi: 10.1016/j.bbih.2022.100436 35469211 PMC9034311

[16] Halstead S Siskind D Amft M Wagner E Yakimov V Shih-Jung Liu Z. Alteration patterns of peripheral concentrations of cytokines and associated inflammatory proteins in acute and chronic stages of schizophrenia: a systematic review and network meta-analysis. Lancet Psychiatry. (2023) 10:260–71. doi: 10.1016/S2215-0366(23)00025-1 36863384

[17] Gilmore JH Fredrik Jarskog L Vadlamudi S Lauder JM. Prenatal infection and risk for schizophrenia: IL-1beta, IL-6, and TNFalpha inhibit cortical neuron dendrite development. Neuropsychopharmacology. (2004) 29:1221–29. doi: 10.1038/sj.npp.1300446 15085088

[18] Ghasemi Noghabi P Shahini N Salimi Z Ghorbani S Bagheri Y Derakhshanpour F. Elevated serum IL-17 A and CCL20 levels as potential biomarkers in major psychotic disorders: a case-control study. BMC Psychiatry. (2024) 24:677. doi: 10.1186/s12888-024-06032-3 39394574 PMC11468266

[19] Li H Peng S Li S Liu S Lv Y Yang N. Chronic olanzapine administration causes metabolic syndrome through inflammatory cytokines in rodent models of insulin resistance. Sci Rep. (2019) 9:1582. doi: 10.1038/s41598-018-36930-y 30733507 PMC6367387

[20] Kelly CW McEvoy JP Miller BJ. Total and differential white blood cell counts, inflammatory markers, adipokines, and incident metabolic syndrome in phase 1 of the clinical antipsychotic trials of intervention effectiveness study. Schizophr Res. (2019) 209:193–7. doi: 10.1016/j.schres.2019.04.021 31118157

[21] Zeng K Wang S Zhang L Zhang Y Ma J. Gender differences in prevalence and associated factors of metabolic syndrome in first-treatment and drug-naïve schizophrenia patients. Ann Gen Psychiatry. (2023) 22:25. doi: 10.1186/s12991-023-00455-0 37381041 PMC10308654

[22] Li Q Chen D Liu T Walss-Bass C de Quevedo JL Soares JC. Sex differences in body mass index and obesity in chinese patients with chronic schizophrenia. J Clin Psychopharmacol. (2016) 36:643–8. doi: 10.1097/JCP.000000000000000594 27811553

[23] Zhu D Montagne A Zhao Z. Alzheimer's pathogenic mechanisms and underlying sex difference. Cell Mol Life Sci. (2021) 78:4907–20. doi: 10.1007/s00018-021-03830-w PMC8720296 33844047

[24] Jarkas DA Villeneuve AH Daneshmend AZB Villeneuve PJ McQuaid RJ. Sex differences in the inflammation-depression link: A systematic review and meta-analysis. Brain Behav Immun. (2024) 121:257–68. doi: 10.1016/j.bbi.2024.07.037 39089535

[25] He J Wei Y Li J Tang Y Liu J He Z. Sex differences in the association of treatment-resistant schizophrenia and serum interleukin-6 levels. BMC Psychiatry. (2023) 23:470. doi: 10.1186/s12888-023-04952-0 37370004 PMC10303764

[26] Lee EE Ancoli-Israel S Eyler LT Tu XM Palmer BW Irwin MR. Sleep disturbances and inflammatory biomarkers in schizophrenia: focus on sex differences. Am J Geriatr Psychiatry. (2019) 27:21–31. doi: 10.1016/j.jagp.2018.09.017 30442531 PMC6489497

[27] Bai YX Luo JX Peng D Sun JJ Gao YF Hao LX. Brain network functional connectivity changes in long illness duration chronic schizophrenia. Front Psych. (2024) 15:1423008. doi: 10.3389/fpsyt.2024.1423008 PMC11221339 38962058

[28] Leucht S Samara M Heres S Davis JM. Dose equivalents for antipsychotic drugs: the DDD method. Schizophr Bull. (2016) 42 Suppl 1:S90–4. doi: 10.1093/schbul/sbv167 PMC4960429 27460622

[29] Kay SR Fiszbein A Opler LA. The positive and negative syndrome scale (PANSS) for schizophrenia. Schizophr Bull. (1987) 13:261–76. doi: 10.1093/schbul/13.2.261 3616518

[30] Si T Yang J Shu L Wang X Kong Q Zhou M. The reliability, validity of PANSS and its implication (in chinese). Chin Ment Health J. (2004) 18:45–7. doi: 10.3321/j.issn:1000-6729.2004.01.016

[31] Kay SR Opler LA Lindenmayer JP. Reliability and validity of the positive and negative syndrome scale for schizophrenics. Psychiatry Res. (1988) 23:99–110. doi: 10.1016/0165-1781(88)90038-8 3363019

[32] Yuan X Yang Q Yao Y Song S Zhou X Liu H. Role of HOMA-IR and IL-6 as screening markers for the metabolic syndrome in patients with chronic schizophrenia: a psychiatric hospital-based cross-sectional study. Eur Arch Psychiatry Clin Neurosci. (2024) 274:1063–70. doi: 10.1007/s00406-023-01618-6 37166483

[33] Cai C Li H Zhang L Li J Duan S Fang Z. Machine learning identification of nutrient intake variations across age groups in metabolic syndrome and healthy populations. Nutrients. (2024) 16:1659. doi: 10.3390/nu16111659 38892592 PMC11175067

[34] Alonso Y Miralles C Algora MJ Valiente-Pallejà A Sánchez-Gistau V Muntané G. Risk factors for metabolic syndrome in individuals with recent-onset psychosis at disease onset and after 1-year follow-up. Sci Rep. (2022) 12:11386. doi: 10.1038/s41598-022-15479-x 35794221 PMC9259625

[35] Zhang R Sun J Wang C Wang X Zhao P Yuan Y. The racial disparities in the epidemic of metabolic syndrome with increased age: A study from 28,049 chinese and american adults. Front Public Health. (2021) 9:797183. doi: 10.3389/fpubh.2021.797183 35178373 PMC8843927

[36] Gao YN Olfson M. National trends in metabolic risk of psychiatric inpatients in the United States during the atypical antipsychotic era. Schizophr Res. (2022) 248:320–8. doi: 10.1016/j.schres.2022.09.023 PMC10135373 36155305

[37] Guan X Chen Y Wang X Xiu M Wu F Zhang X. Total antioxidant capacity, obesity and clinical correlates in first-episode and drug-naïve patients with schizophrenia. Schizophr Res. (2024) 264:81–6. doi: 10.1016/j.schres.2023.12.004 38113675

[38] Mezquida G Savulich G Garcia-Rizo C Garcia-Portilla MP Toll A Garcia-Alvarez L. Inverse association between negative symptoms and body mass index in chronic schizophrenia. Schizophr Res. (2018) 192:69–74. doi: 10.1016/j.schres.2017.04.002 28412089

[39] Sicras-Mainar A Maurino J Ruiz-Beato E Navarro-Artieda R. Prevalence of metabolic syndrome according to the presence of negative symptoms in patients with schizophrenia. Neuropsychiatr Dis Treat. (2015) 11:51–7. doi: 10.2147/NDT.S75449 PMC4283985 25565850

[40] Soontornniyomkij V Lee EE Jin H Martin AS Daly RE Liu J. Clinical correlates of insulin resistance in chronic schizophrenia: relationship to negative symptoms. Front Psychiatry. (2019) 10:251. doi: 10.3389/fpsyt.2019.00251 31065243 PMC6488983

[41] Dong S Schneider-Thoma J Bighelli I Siafis S Wang D Burschinski A. A network meta-analysis of efficacy, acceptability, and tolerability of antipsychotics in treatment-resistant schizophrenia. Eur Arch Psychiatry Clin Neurosci. (2024) 274:917–28. doi: 10.1007/s00406-023-01654-2 PMC11127860 37526675

[42] Dodd K Legget KT Cornier M-A Novick AM McHugo M Berman BD. Relationship between functional connectivity and weight-gain risk of antipsychotics in schizophrenia. Schizophr Res. (2024) 267:173–81. doi: 10.1016/j.schres.2024.03.033 PMC11332974 38552340

[43] Carruzzo F Kaiser S Tobler PN Kirschner M Simon JJ. Increased ventral striatal functional connectivity in patients with schizophrenia during reward anticipation. NeuroImage Clin. (2022) 33:102944. doi: 10.1016/j.nicl.2022.102944 35078045 PMC8789684

[44] Salehi MA Zafari R Mohammadi S Shahrabi Farahani M Dolatshahi M Harandi H. Brain-based sex differences in schizophrenia: A systematic review of fMRI studies. Hum Brain Mapp. (2024) 45:e26664. doi: 10.1002/hbm.26664 38520370 PMC10960555

[45] Lee R Griffiths SL Gkoutos GV Wood SJ Bravo-Merodio L Lalousis PA. Predicting treatment resistance in positive and negative symptom domains from first episode psychosis: Development of a clinical prediction model. Schizophr Res. (2024) 274:66–77. doi: 10.1016/j.schres.2024.09.010 39260340

[46] Tian Y Wang D Wei G Wang J Zhou H Xu H. Prevalence of obesity and clinical and metabolic correlates in first-episode schizophrenia relative to healthy controls. Psychopharmacol (Berl). (2021) 238:745–53. doi: 10.1007/s00213-020-05727-1 33241480

[47] Brand BA Sommer IE Gangadin SS Tanskanen A Tiihonen J Taipale H. Real-world effectiveness of menopausal hormone therapy in preventing relapse in women with schizophrenia or schizoaffective disorder. Am J Psychiatry. (2024) 181:893–900. doi: 10.1176/appi.ajp.20230850 39262210

[48] Pahwa R Singh A Adams-Huet B Devaraj S Jialal I. Increased inflammasome activity in subcutaneous adipose tissue of patients with metabolic syndrome. Diabetes Metab Res Rev. (2021) 37:e3383. doi: 10.1002/dmrr.3383 32652811

[49] Sirota P Hadi E Djaldetti M Bessler H. Difference in inflammatory cytokine production by mononuclear cells from obese and non-obese schizophrenic patients. Acta Psychiatr Scand. (2015) 132:301–5. doi: 10.1111/acps.12396 25627461

[50] Américo-Da-Silva L Aguilera J Quinteros-Waltemath O Sánchez-Aguilera P Russell J Cadagan C. Activation of the NLRP3 inflammasome increases the IL-1β Level and decreases GLUT4 translocation in skeletal muscle during insulin resistance. Int J Mol Sci. (2021) 22:10212. doi: 10.3390/ijms221910212 34638553 PMC8508423

[51] Hofwimmer K de Paula Souza J Subramanian N Vujičić M Rachid L Méreau H. IL-1β promotes adipogenesis by directly targeting adipocyte precursors. Nat Commun. (2024) 15:7957. doi: 10.1038/s41467-024-51938-x 39261467 PMC11390900

[52] Torres SV Man K Elmzzahi T Malko D Chisanga D Liao Y. Two regulatory T cell populations in the visceral adipose tissue shape systemic metabolism. Nat Immunol. (2024) 25:496–511. doi: 10.1038/s41590-024-01753-9 38356058

[53] Varra F-N Varras M Varra V-K Theodosis-Nobelos P. Molecular and pathophysiological relationship between obesity and chronic inflammation in the manifestation of metabolic dysfunctions and their inflammation-mediating treatment options (Review). Mol Med Rep. (2024) 29:95. doi: 10.3892/mmr.2024.13219 38606791 PMC11025031

[54] Fang X Wang Y Chen Y Ren J Zhang C. Association between IL-6 and metabolic syndrome in schizophrenia patients treated with second-generation antipsychotics. Neuropsych Dis Treat. (2019) 15:2161–70. doi: 10.2147/NDT.S202159 PMC6681158 31534339

[55] Bosia M Spangaro M Sapienza J Martini F Civardi S Buonocore M. Cognition in schizophrenia: modeling the interplay between interleukin-1β C-511T polymorphism, metabolic syndrome, and sex. Neuropsychobiology. (2021) 80:321–32. doi: 10.1159/000512082 33395686

[56] Imano N Shojima K Tamaki K Shinmura K. Estrogen contributes to the sex difference in the occurrence of senescence-related T cells during the development of visceral adipose tissue inflammation. Am J Physiol Heart Circ Physiol. (2023) 324:H662–74. doi: 10.1152/ajpheart.00469.2022 36930655

[57] Morishita M Miyagi M Iwamoto Y. Effects of sex hormones on production of interleukin-1 by human peripheral monocytes. J Periodontol. (1999) 70:757–60. doi: 10.1902/jop.1999.70.7.757 10440637

[58] Patlola SR Donohoe G McKernan DP. Anti-inflammatory effects of 2nd generation antipsychotics in patients with schizophrenia: A systematic review and meta-analysis. J Psychiatr Res. (2023) 160:126–36. doi: 10.1016/j.jpsychires.2023.01.042 36804109

[59] Zhao T Zhang K Zhang Y Yang Y Ning X Hu Y. Do proinflammatory cytokines play a role in clozapine-associated glycometabolism disorders? Psychopharmacol (Berl). (2021) 238:1979–90. doi: 10.1007/s00213-021-05824-9 PMC8233252 33774704

